# The interplay between BDNF and PGC-1 alpha in maintaining brain health: role of exercise

**DOI:** 10.3389/fendo.2024.1433750

**Published:** 2024-08-22

**Authors:** Xuecui Bi, Jing Fang, Xin Jin, Anand Thirupathi

**Affiliations:** ^1^ Institute of Physical Education and Training, Capital University of Physical Education and Sports, Beijing, China; ^2^ Basic Department, Dezhou Vocational and Technical College, Dezhou, China; ^3^ International Department, Beijing No.35 High School, Beijing, China; ^4^ Faculty of Sports Science, Ningbo University, Ningbo, China

**Keywords:** BDNF, PGC-1 alpha, brain health, exercise, molecular signaling

## Abstract

Throughout our evolutionary history, physical activity has played a significant role in shaping our physiology. Advances in exercise science have further reinforced this concept by highlighting how exercise can change gene expression and molecular signaling to achieve various beneficial outcomes. Several studies have shown that exercise can alter neuronal functions to prevent neurodegenerative conditions like Parkinson’s and Alzheimer’s diseases. However, individual genotypes, phenotypes, and varying exercise protocols hinder the prescription of exercise as standard therapy. Moreover, exercise-induced molecular signaling targets can be double-edged swords, making it difficult to use exercise as the primary candidate for beneficial effects. For example, activating PGC-1 alpha and BDNF through exercise could produce several benefits in maintaining brain health, such as plasticity, neuronal survival, memory formation, cognition, and synaptic transmission. However, higher expression of BDNF might play a negative role in bipolar disorder. Therefore, further understanding of a specific mechanistic approach is required. This review focuses on how exercise-induced activation of these molecules could support brain health and discusses the potential underlying mechanisms of the effect of exercise-induced PGC-1 alpha and BDNF on brain health.

## Introduction

While advancement in the sciences increases the longevity of people, it has also challenged discoveries in the medical field witnessed by several non-communicable diseases such as diabetes, hypertension, obesity, neurodegeneration, and cancer. Indeed, all these non-communicable diseases contribute to mortality rates of up to 80 percent in certain developing countries and are considered to be the number one killer around the world (1, 2). However, all these diseases are somehow attributed to less or without any physical activity, which fails to adapt or sustain the body to fight against these diseases. The physically inactive people ratio has been increasing globally, according to the World Health Organization (WHO), showing physical inactivity can be the current or future pandemic and can make this issue the most important public health priority (3). It is well established that a proper diet with moderate physical activity can reverse most of these non-communicable diseases, representing exercise as a promising health strategy for decreasing these diseases. It has been recommended that children and adults perform at least 150-300 minutes of moderate exercise or 75-150 minutes of vigorous exercise in a week to reverse these health effects (4). Nevertheless, it is also recommended that adults perform strength exercises with a moderate intensity 2 to 3 times a week (4). After all, these forms of exercise could contribute to activating certain molecular signaling that ultimately produces longevity benefits and prevents these non-communicable diseases. This review narrows the way of discussing general molecular pathways and molecules involved in these pathways into more specific molecular targets, such as brain-derived neurotrophic factor (BDNF) and peroxisome proliferator-activated receptor gamma coactivator 1-alpha (PGC-1 alpha), in maintaining brain health.

Research has shown that moderate exercise has multiple benefits for maintaining brain health, especially for improving neuronal plasticity, cell survival, and cognitive functions (5). However, the effects of exercise reported by these studies are minimal, and better understandable mechanisms are required to bridge the gaps in our knowledge so far and pave the way to designing exercise regimens for brain health. For example, studies reported so far for improving brain health and cognition are based on pre-and post-exercise, which do not take BDNF and PGC-alpha as the primary targets. Therefore, presenting how pre- and post-exercise can potentially modulate the BDNF and PGC-alpha to improve brain health could reveal BDNF and PGC-alpha as therapeutical targets for improving brain health.

As mentioned, exercise is one of the major nontherapeutic methods in maintaining brain health. For example, endurance training (treadmill running) improves neurogenesis in the hippocampal area (6), while resistance training can increase the proliferation of neural cells and neurogenesis (7). We systematically analyzed the available results in different databases, including PubMed, Web of Science, and Google Scholar, focusing on articles published in the last two decades related to physical activity, physical exercise, brain health, BDNF, and PGC-1 alpha. The search strategy involved a combination of keywords and abstracts (MeSH terms) related to physical activity, physical exercise, BDNF, and PGC-1 alpha. These keywords were used with the boolean operators (AND and OR) to identify articles that directly addressed the link between physical exercise and BDNF and PGC-1 alpha in brain health.

## Pleiotropic effects of BDNF

The novel neurotrophic factor called BDNF was identified in 1982 to support the survival and growth of chick embryo dorsal ganglia (8). Since then, several studies have been reported on different aspects of BDNF in the central nervous system, including its role in neuronal development and synaptic plasticity (9–11), indicating the role of BDNF as a key regulator in the learning and memory (12). Although these studies show the importance of BDNF in brain physiology and pathology, its multifunctional role with various signaling pathways in the larger aspects still fails to formulate specific hypotheses or directions for future research, which is a daunting challenge in this field (13). Therefore, additional studies that specifically stimulate the activation of BDNF or its upstream targets, which can activate BDNF, could take BDNF research to the next level.

BDNF is a single molecule that could transiently activate multiple signaling based on its expression, concentration, and release site. Physical exercise can also activate this molecule or its upstream targets to activate BDNF exclusively (13, 14). For instance, exercise-induced activation of PGC-1 alpha, a mitochondrial biogenesis protein (15, 16), could mediate this BDNF activation. This can contribute to neuronal health by reducing ROS concentration and oxidative stress and increasing neuronal survival. Nevertheless, it is unclear whether PGC-1 alpha-induced biogenesis is the major reason for all these BDNF-induced benefits in the brain. Evidence suggests that PGC-1 alpha promotor activation can increase BDNF levels in the hippocampal region (17, 18). This may be due to the activation of extracellular signal-regulated kinases (ERKs) and cAMP response element-binding protein (CREB), which mediate the activation of PGC-1 alpha by BDNF (18–20), while BDNF-activated PGC-1 alpha, nuclear respiratory factor 1/2 (NRF1/2), and mitochondrial transcription factor TFAM can increase mitochondrial biogenesis and synapse formation in the hippocampal neurons (18). However, there is currently no comprehensive review of how exercise-induced PGC-1 alpha regulates the beneficial effects of BDNF in the central nervous system.

## Exercise role on BDNF downstream signaling for brain health

**Figure 1:**
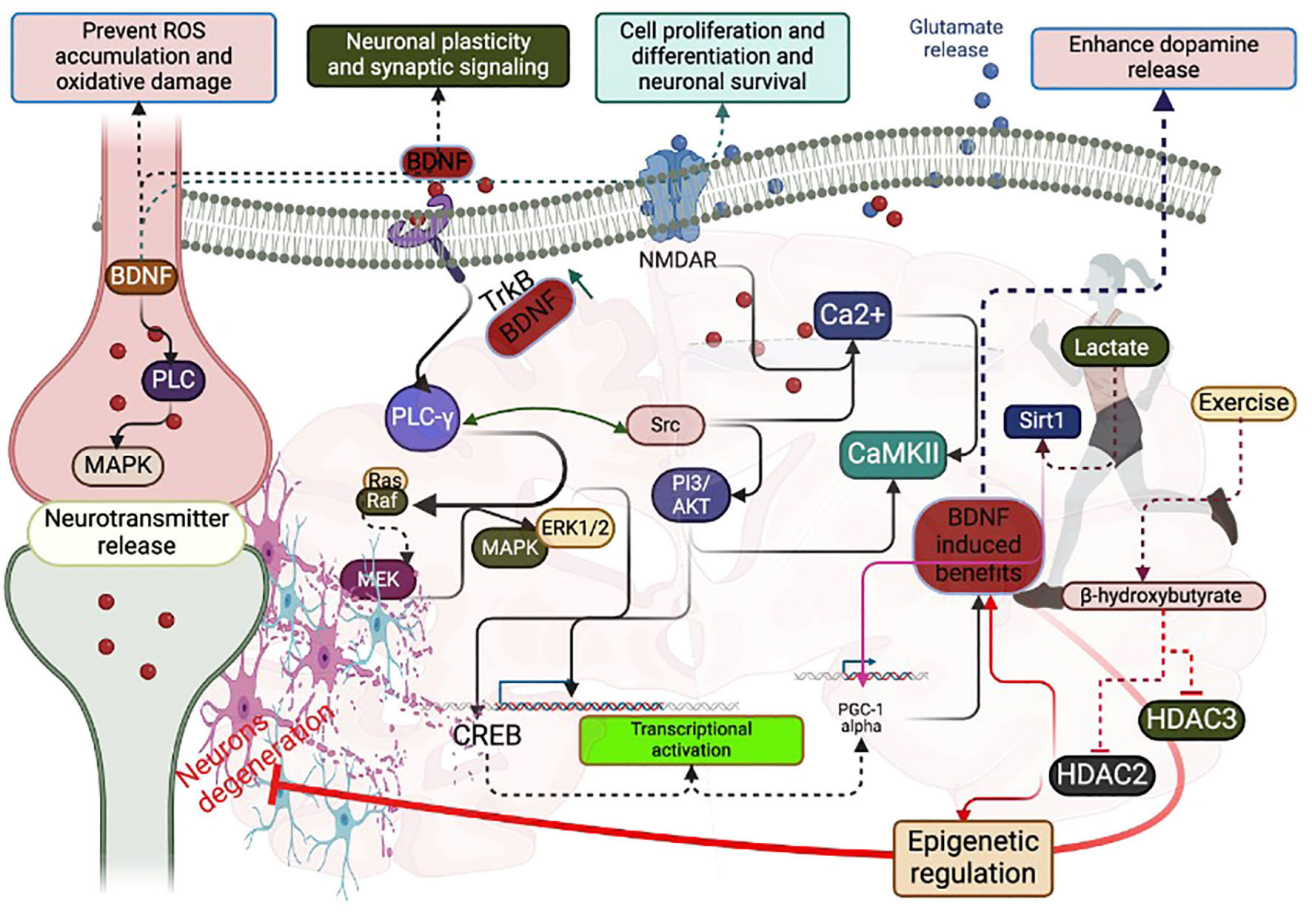
Possible signaling pathways that interfere with BDNF and PGC-1 alpha during exercise: Exercise-induced lactate increases BDNF concentration mediated by Sirt1, PGC-1 alpha, and FNDC5. Exercise-induced beta-hydroxybutyrate decreases HDAC2 and HDAC3 for epigenetic regulation, favoring BDNF levels. CREB activation activates PGC-1 alpha to increase BDNF concentration. Exercise-induced phosphorylation of PLC-γ increases BDNF-induced glutamate release and Ca2+ flux. Exercise-induced phosphorylation of PLC-γ activates CREB through PI3/AKT, Ras/Raf, MEK/MAPK/ERK1/2. CREB can activate PGC-1 alpha to increase BDNF.

Both pro and matured BDNF proteins are expressed at higher levels in the hippocampal and cortex regions of the brain. Exercise could influence the transport of BDNF within the axon terminal and dendritic compartments. However, whether exercise can initially activate pro or matured BDNF for signaling activities is unknown. For instance, an exercise-induced (both aerobic and resistance with high-intensity) increase in lactate can directly activate the matured BDNF in the brain, either directly or through enzymatical cleavages of pro-BDNF (21–23). This could further activate the BDNF-induced signaling. For example, treadmill exercise once a week for two weeks can increase the BDNF-mediated N-methyl-D-aspartate (NMDA) receptor expression to improve neuronal plasticity and synaptic signaling through its ionotropic properties (24–26). Further, running exercise increases the activation of glutamate receptors to improve mental health (27). Furthermore, voluntary wheel-running exercise-induced activation of tyrosine receptor kinase B (TrkB) by BDNF enhanced dopamine release, possibly triggering the many downstream pathways for improving neuronal functions and survival (28). For example, TrkB activation can lead to activate the Src homolog domain 2 (SH2) and phosphorylation of the Phospholipase C-γ (PLC-γ), phosphatidylinositol 3 kinase/mechanistic target of rapamycin (PI3K/mTOR) activation, and mitogen-activated protein kinase/extracellular signal-regulated kinase (MAPK/ERK) pathways (29–31) (**Figure 1**). All of these signals are linked to the improvement of synaptic plasticity. For example, 5 weeks of aerobic exercise programs decreased the phosphorylation of PLC-γ to normalize the BDNF upregulation and glial hyperactivity in neuropathic pain (32). In addition, exercise-induced activation of PLC-γ pathway could activate the PI3k/Akt and Ras/Erk1/2 signaling, which are responsible for survival and regeneration (33). Also, exercise-induced activation of the PLC-γ pathway can support calcium release by activating calcium-dependent protein kinases for regulating synaptic plasticity and proliferation and differentiation. Swimming exercise can increase the activation of TrkB receptor and BDNF (34), which could activate the MAPK and PLC-γ pathways to influence the presynaptic neurotransmitter release.

## The interplay between BDNF and PGC-1 alpha for maintaining brain health-role of exercise

**Figure 2:**
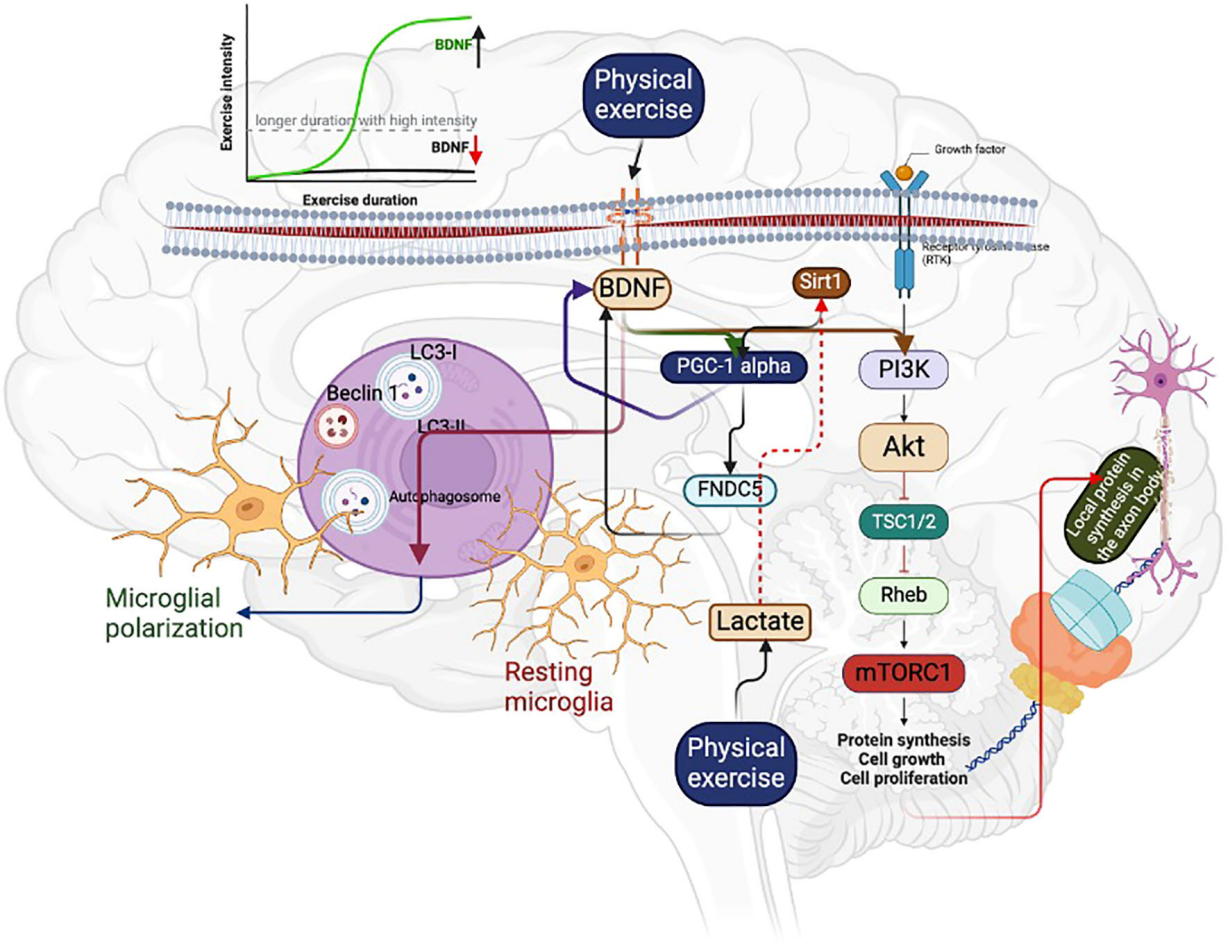
Regular physical exercise can increase BDNF levels, which can activate PGC-1 alpha and FNDC5. This can lead to the activation of the mTOR signaling pathway, resulting in an increase in local protein synthesis in the axon body. Additionally, BDNF can induce autophagy, which causes microglial polarization. This helps to remove damaged neurons. However, it is important to note that high-intensity exercise with longer duration can decrease BDNF concentration, as indicated in the graphical representation. On the other hand, acute exercise with high intensity can increase the BDNF level, as shown by the green mark.

As mentioned, BDNF regulates cell proliferation, differentiation, and survival in brain physiology and pathology. For example, activating protein synthesis signaling, such as the mammalian target of rapamycin (mTOR), by BDNF could increase translation and local protein synthesis within the axon body. Physical exercise (treadmill exercise for 2 weeks for 20 mins a day for five days/week) is the obvious activator of protein synthesis signaling, including AKT/mTOR by BDNF-mediated autophagy, resulting in microglia polarization after the peripheral nerve injury, evidenced by the higher expression of autophagy markers such as LC3-II/LC3-I and Beclin1 (35–37) (**Figure 2**). This may be due to the PGC-1 alpha-mediated BDNF that could cause the microglial M1/M2 polarization and autophagy flux to prevent neurotoxicity (38). However, this effect depends on the dose-response relationship between exercise protocols and BDNF concentration or PGC-1 alpha levels. For instance, acute exercise for 15 minutes and 4 hours of rowing can increase BDNF concentration, while long-term sprint exercise can decrease BDNF levels (39). These findings suggest that exercise protocols can significantly influence the concentration of BDNF, especially for longer periods with higher intensity, which can negatively influence BDNF levels (**Figure 2**). In addition to PGC-1 alpha, running exercise can activate other transcription factors like cAMP response element-binding protein (CREB), increasing BDNF expression and promoting hippocampal neuron survival (40). For example, running exercise doubles the CREB level in the hippocampus, and it can be kept increasing for at least a week (41). Other exercise-induced signaling, such as insulin-like growth factor-1 and nitric oxide/cGMP, can crosstalk for increasing CREB expression, resulting in higher levels of BDNF in the neurons (42). Further, maintaining synaptic plasticity requires local protein synthesis; exercise-induced BDNF could compensate for the local protein synthesis by activating mTOR signaling (43–46). A study has reported that BDNF can regulate the transport of mRNAs and their translations in the synapses by altering the initiation and elongation phase of protein synthesis (47). This can help in the long-term alteration of the synaptic proteome (47). In addition, mTOR is involved in neuronal development, synaptic plasticity, and cognition (48), and any deregulation in the mTOR signaling can be involved in brain pathologies such as Parkinson’s disease and Alzheimer’s disease. Studies have shown that exercise with different protocols (treadmill running) regulates the mTOR signaling (35, 44), which could contribute to more specific functions in the brain, including axonal sprouting and regeneration and myelination and dendritic spine growth (48). Moreover, mTOR regulates the mitochondrial function in the neuronal cell through PGC-1 alpha by reducing the accumulation of ROS in the neuronal cell. Running exercises for 90 days could activate the PGC-1 alpha and other protein expressions, including fibronectin type III domain-containing protein 5 (FNDC5) and mTOR, extracellular signal-regulated kinases (ERK), and sirtuin 1 (SIRT1) in the hippocampal (49). The possible mechanism for these protein expressions in the hippocampus may be due to the expression of PGC 1 alpha, which might regulate FNDC5/BDNF (50) (**Table 1**).

**Table 1:** Exercise protocols that interferes the BDNF and PGC-1 alpha-induced signaling pathways in brain health.

Exercise types	Duration and protocols	Pathways that interfere BDNF and PGC-1 alpha	Significance	References
Acute aerobic exercise	Two 30-min exercise bouts (cycle ergometer)	Lactate induced activation of BDNF is mediated by SIRT1, PGC-1 alpha and FNDC5.	Neurological and cognitive improvements and learning memory.	(51)
Motorized treadmill for 30 min once a day for 2 weeks	Exercise is running at a speed of 2 meters/min for the first 5 min, 5 meters/min for the next 5 min, and 8 meters/min for the last 50 min	Treadmill exercise induced NMDA receptor decrease the neuronal apoptosis	Improve neuronal plasticity and synaptic signaling through its ionotropic properties and inhibit the neuronal apoptosis.	(52)
voluntary wheel-running exercise	30 days	TrKB and BDNF increase could enhance the dopamine release in striatum	Exercise induced BDNF could enhance the dopamine release in neuropsychiatric disorders, including Parkinson's, depression, and anxiety.	(28)
Swimming exercise	Once a day for 5 days for 5 weeks (Animal study)	Exercise induced phosphorylation of PLC-γ increase the BDNF induced glutamate release. BDNF and other cellular signaling such as nitric oxide and kinase pathways such as PKA, ERK and PLCγ-1 can increase the CREB phosphorylation.	PLC-γ phosphorylation can improve the recovery of mechanical allodynia after nerve lesion. Converging BDNF mediated signaling can improve in neuropathic pain.	(32)
Swimming exercise (Animal studies)	Swimming exercise was consist of 15 min/day, 5 days/week, to begin their exercise training for first two weeks. Next duration was extendend upto 20 min from 3rd week and 30 mins during the 4th to 12th weeks.	This exercise protocol could activate BDNF/TrkB survival signaling such as MEK/MAPK/ERK, PI3K/AKT, and Bcl2-family survival pathways.	Activation of BDNF induced survival pathways decrease the neural apoptosis.	(53)
treadmill exercise	Treadmill exercise training was performed at 12 m/min, 60 min/day, 5 days/week for 3 months.	Treadmill exercise could increase BDNF, PI3-k/Akt pathway and HSP70 for improving Aβ-induced cognitive dysfunction	Improve the cognitive dysfunction and neuronal survival.	(54)
running wheel exercise	Voluntary exercise for 4 weeks	Exercise induced increase of β-hydroxybutyrate decreases the HDAC2 and HDAC3 to increase the BDNF	Changes in the epigenetic landscape to promote BDNF expression and exercise induced β-hydroxybutyrate increase the neurotransmitter release via TrkB receptor.	(55)
Running exercise	30 days of free- running wheel exercise	Running exercise activate the PGC-1 alpha mediated BDNF through FNDC5	Reducing the accumulation of ROS and oxidative damage in the neuronal cell and improve the mitochondrial function.	(49)

Other signaling proteins, such as SIRT1, can also regulate the PGC-1 alpha by exercise. For example, swimming could reduce neuroinflammation through SIRT1-mediated pathways, including PGC-1 alpha, and improve cognitive functions (56). Furthermore, NMDAR plays a crucial role in neuronal communication, and treadmill running exercise regulates the NMDAR by BDNF expression for regulating dendritic regeneration, which can contribute to decrease the neurologic and psychiatric disorders (25). In addition, other studies have shown that exercise-induced activation of NMDAR could induce motor neuron protection and learning capacity (57, 58). Exercise plays a significant role in activating Dexras1, essential for promoting hippocampal neurogenesis and improving cell survival. Inhibition of Dexras1, on the other hand, can interfere with the exercise-dependent activation of ERK/MAPK and CREB, abolish NMDAR upregulation, and inhibit BDNF in the dentate gyrus (59). Therefore, the activation of Dexras1 through exercise is crucial for maintaining healthy brain function. In addition, other PGC- 1 alpha stimulating factors, such as nitric oxide and estrogen, could be positively regulated by exercise (60–63), and this could be helpful to increase the mitochondrial biogenesis through PGC- 1 alpha for restoring synaptic functions in neurodegenerative diseases such as Parkinson`s and Alzheimer’s diseases (18). Nevertheless, further research is required to determine whether these factors can upregulate the BDNF through PGC-1 alpha.

## Effect of environmental enrichment and physical exercise on BDNF response

An enriched environment naturally influences brain morphology, molecular signaling, and behavior (64, 65). This effect has primarily been observed in laboratory settings (66). However, an excessively enriched environment can also negatively impact brain functions due to the stress it imposes on the organism, resulting in reduced sensorimotor stimulation (67). A recent study has demonstrated that a combination of physical exercise and an enriched environment yields superior effects on the brain by activating molecules such as BDNF/TrkB (68). For example, combining an enriched environment and swimming exercise improved the BDNF/TrkB response more than either the enriched environment or swimming alone, suggesting the effectiveness of the combined approach (69). A study has shown that treadmill running for 40 mins for 6 days at a moderate intensity in an enriched environment improved learning and memory through elevating BDNF/TrkB (70). This is mainly due to rearranging novel objects to induce a fresh, enriched environment (70). At the molecular level, PGC-1 alpha could be the main trigger for BDNF response under an enriched environment and exercise. A study has shown that an enriched environment increased cognitive impairment in the aged offspring by reducing neuroinflammation and oxidative stress through elevating Sirt1/PGC-1 alpha and FNDC5, which can overlap the BDNF signaling for its elevation. These BDNF-induced cognitive improvements are due to neural plasticity, including BDNF-induced neurogenesis and BDNF-caused structural modification and molecular changes. For example, elevation of BDNF response due to an enriched environment increases the neurogenesis through pCREB, stromal-cell derived factor-1, and C-X-C chemokine receptor type 4 and improves cognition (70). In addition, enriched environment influences the epigenetic landscape by affecting the BDNF gene methylation. For example, enriched environment reduced the exon IV expression by reversing histone methylation, which is linked with the pathophysiology of depression (71). Physical exercise has also been linked with the alteration of the epigenetic landscape, and studies have shown that exercise decreases BDNF methylation and increases BDNF mRNA, which increases learning and memory (72). However, it is important to recognize that these effects should be further studied in an enriched environment and under physical exercise conditions.

## Conclusion and future directions

Physical exercise has several benefits for promoting a healthy body and mind at any age. This can claim that exercise is one of the nonpharmacological therapies that delays the negative effects of physiological aging and pathological neurodegeneration on brain health. The exercise protocols used to achieve these benefits in rodents and humans have varied in terms of types, intensity, duration, and individual phenotypes and genotypes. While exercise is known to maintain physical and mental health, it is unclear whether it should be seen as an enjoyable activity or as a drug or therapy to influence brain health. Further studies are needed to determine how different exercise protocols, whether performed before, during, or after exercise, can affect molecular signaling in the brain. Specifically, researchers need to investigate how individual molecules or proteins activated by exercise can influence downstream molecules in molecular signaling and contribute to brain health. For example, a single molecule activated by exercise can synergistically impact the entire brain (57), such as BDNF, which can activate various downstream targets to improve plasticity, proliferation, differentiation, and cell survival while helping release multiple neurotransmitters (55, 57). Other pharmacological approaches, such as gene therapy, can also increase BDNF. However, the vector toxicity used in this method can cause protein instability and tumor formation in the local neuron, mainly; protein instability can increase BDNF expression overly and may produce deleterious effects on neuronal circuits, learning, and memory (73). To circumvent this issue, engineered cell grafts can be used to increase BDNF expression, which can improve motor performance and decrease striatal neuron damage (74). Nevertheless, graft rejection may be a risk factor in this approach. Other small molecules, such as drugs (ampakines, memantine, and riluzole), significantly enhance the expression and release of BDNF to treat Alzheimer’s and Parkinson’s diseases (75, 76). In addition, approaches like intermittent fasting, enriched environment, and physical exercise can also increase the BDNF response (76). Exercise-induced activation of PGC-1 alpha can regulate BDNF expression to bring about the benefits of both BDNF and PGC-1 alpha, such as increasing mitochondrial biogenesis and decreasing ROS formation and oxidative damage. However, it still needs to be determined whether exercise activates these molecules simultaneously or activation of one molecule leads to the activation of others, and further investigation is necessary in this regard.

Certain groups cannot perform physical exercise for various reasons, such as aging, undergoing a rehabilitation program, or having motor function impairment. Recently, virtual reality-based exercise programs have been developed to support these individuals. Further research is needed to investigate how molecular changes occur during virtual exercise programs. If these types of exercise programs prove to be effective, they could be particularly useful for people who are unable to perform real-time exercise programs. Designing exercise programs tailored to each individual’s needs could potentially overcome people’s genetic and epigenetic differences. This could be particularly helpful in understanding the benefits of exercise on the nervous system, considering that each individual’s learning system is unique and may cause frequent changes in the genetics and epigenetics of the brain. In particular, studying the expression of exercise-induced genes such as PGC-1 alpha and BDNF during or after exercise could provide insights into the specific benefits of these molecular targets for brain health.
